# A method for measuring rotation of a thermal carbon nanomotor using centrifugal effect

**DOI:** 10.1038/srep27338

**Published:** 2016-06-02

**Authors:** Kun Cai, Jingzhou Yu, Jiao Shi, Qing H. Qin

**Affiliations:** 1 College of Water Resources and Architectural Engineering, Northwest A&F University, Yangling 712100, China; 2 Research School of Engineering, the Australian National University, Canberra, ACT, 2601, Australia

## Abstract

A thermal nanomotor is relatively easy to fabricate and regulate as it contains just a few or even no accessory devices. Since the double-wall carbon nanotube (CNT)-based rotary nanomotor was established in a thermostat, assessment of the rotation of the rotor (inner tube) in the stator (outer tube) of the nanomotor has been critical, but remains challenging due to two factors: the small size of the rotor (only a few nanometers) and the high rotational frequency (»1 GHz). To measure the rotation of the nanomotor, in the present study, a probe test method is proposed. Briefly, the rotor is connected to an end-tube (CNT) through a graphene (GN) nanoribbon. As the CNT-probe is on the trajectory of the end-tube which rotates with the rotor, it will collide with the end-tube. The sharp fluctuation indicating the probe tip deflection can be observed and recorded. As a curly GN by hydrogenation is adopted for connecting the rotor and the end-tube, collision between the end-tube and the probe tip occurs only when the centrifugal force is higher than a threshold which can be considered as the rotational frequency of the rotor being measured by the present method.

With the rapid development of nanofabrication technology, smaller nano-electromechanical systems (NEMS)^1^,^2^,^3^,^4^ are being developed from the earlier concept design. Among such nanodevices, nanomotors have potential applications in practical engineering as actuators^5^,^6^, sensors^7^,^8^, biomedical nanodevices^9^,^10^, etc. In fabricating such nanodevices, carbon nanostructures^2^,^11^ play an important role due to their excellent mechanical properties (high modulus + intershell lower friction), which have made possible realization of linear bearings from multi-wall carbon nanotubes^12^. Specifically, carbon nanotubes (CNT) have been used to fabricate nano-oscillators^13^, nanobearings/nanomotors^14^,^15^,^16^, nanocages from graphene^17^, etc.

CNT-based rotary nanomotors have attracted significant attention from researchers. For instance, Fennimore^18^ built a synthesized NEMS with a rotatable metal plate on a CNT bearing. In the system they developed, a metal plate was driven to rotate on the CNT under the external electric field. On a different basis from the electricity-driven motor^19^,^20^,^21^, Barreiro *et al*.^14^ in 2008 fabricated a nano cargo system from multi-wall carbon nanotubes (MWCNT) driven by the thermal gradient along the axis of the tubes. The rotation and translational motion of the outer tube were found to bond with a mass along the tube axis. Using a molecular dynamics simulation method, Zambrano *et al*.^15^ studied the motion of a short inner tube in a longer outer tube with different temperatures at each end. More detail as to the effect of thermal gradient on the motion of the double-wall carbon nanotube motor can be found in ref. 16.

Compared to motors driven by different physical fields, e.g., electricity, temperature and velocity of gas/liquid flow^22^,^23^, the temperature motor is the simplest method for fabrication and regulation of a nanomotor. This simplicity has motivated the development of temperature controlled nanomotor. Recently, we^24^ found that DWCNTs can form an autonomous motor in a thermostat. In our model, the outer tube (stator) was fixed and the free inner tube was actuated to rotate at a high rotational frequency (>100 GHz) when the geometrical symmetry of the stator was lost^25^. In that study, the thermal motor was only few nanometers in diameter. Due to the factors of small size and high rotational speed, the rotation of the rotor is difficult to measure using existing *in situ* methods. To ascertain the occurrence of rotation of the nanomotor, we suggest a probe test method in this report. The hypothesis is that, if we bond a wing made from graphene and a CNT on the rotor (Fig. 1), the wing will be driven to rotate with the rotor by the stators. As the rotational frequency increases to a very high value, the velocity of the end-tube also has a very high value and the moment of the end-tube will become large enough to impact the probe tip with an obvious deflection. The deflection of the probe tip reflects the rotation of the rotor.

Based on this concept, a series of numerical experiments on the dynamic responses of the system are conducted. Two major factors are considered: one is the distance between the probe tip and the axis of the rotor, a feature that has a significant influence on the interaction between the end-tube and the probe tip; the other is the initial configuration of the wing (GN + end-tube), e.g., a flat GN rotates with the rotor, or a GN is wound onto the end-tube by hydrogenation on one side of the GN near the end-tube.

## Model and Method

**Figure 1:**
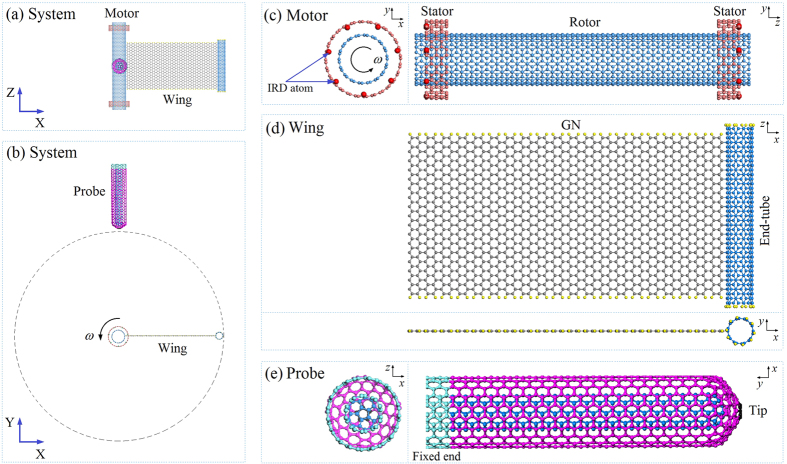
Schematics of system shown in (**a**,**b**) containing three components for measuring rotation of the rotary motor driven by thermal vibration of end carbon atoms. XYZ is the global coordinate system. “*xyz*” is the local coordinate system of each component. Axial directions of either X-*x*, Y-*y* or Z-*z* are parallel. (**c**) The thermal motor (component 1) is a DWCNT system, in which the inner tube (9, 9) acts as a rotor and the outer tubes (14, 14) are fixed as two stators. Each stator has 7 (red) atoms with inward radial deviation (IRD) of 0.4 times 0.142 nm, i.e., ~0.057 nm. (**d**) The wing (component 2) is bonded with the inner tube in the motor. The wing itself is fabricated by combining a GN with a (5,5) CNT. Each boundary carbon atom on the wing is bonded with a hydrogen atom (yellow). The wing rotates together with the (9, 9) rotor in the motor at high temperature, e.g., 300 K. The dashed circle with radius of ~9.950 nm is the external boundary of the wing rotating at its initial size. (**e**) The probe (component 3) is a one-end-capped DWCNT (5,5)/(10,10). The capped end is the tip of the probe. The other end is fixed during simulation. In (**b**), the gap between the probe tip and the dashed circle has an effect on the dynamic response of the components. The initial value of the gap is + 0.142 nm. Hence, the distance between the probe tip and the axis of the rotor is ~10.092 nm.

The dynamic response of the system shown in Fig. 1(a,b) is investigated using the open source molecular dynamic simulation package LAMMPS^26^,^27^. To derive a precise description of the interaction among carbon and/or hydrogen atoms, AIREBO^28^ potential is adopted. That potential is popular in carbon/hydrogen system simulation studies^17^,^21^,^29^,^30^,^31^,^32^. The potential contains three parts, REBO, Lennard-Jones (L-J), and torsion. The cut-off of the L-J potential is three times 0.34 nm.

The testing system consists of three components or six parts, and their parameters are listed in Table 1. Before simulation, energy minimization for the system is carried out using the steepest descending algorithm. After minimization, the carbon atoms on the stators are fixed and the light blue carbon atoms on the four circular lines of the end of probe are fixed (Fig. 1(b,e)). The rest of the system, i.e., the rotating part (i.e., rotor + wing) and the probe, is at canonical NVT ensemble with T = 300 K. The time step for integration is 0.001 ps.

## Results and Discussion

### Comparison of dynamic responses of different models

**Figure 2:**
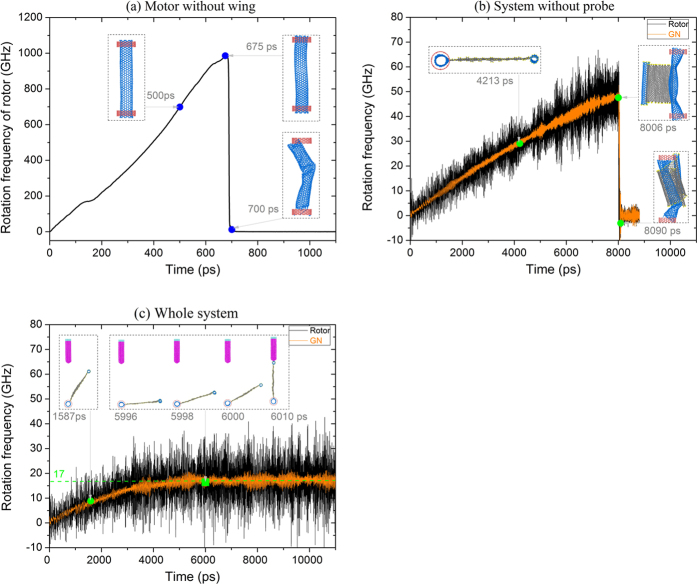
Models comparison. (**a**) Rotational frequency of rotor without wing. (**b**) Rotational frequency of rotor and wing without probe. (**c**) Rotational frequency of rotor and wing when probe is present.

Figure 2 presents the rotational histories of the rotating part in the motor. The rotational acceleration processes are different among the three models. In Fig. 2a, the rotational frequency of the rotor without a wing increases rapidly from zero to the maximal value of 987 GHz after ~675 ps acceleration by the stators. The rotational acceleration is clearly determined by three factors. The first is the IRD^25^ of the end carbon atoms on the stators. It is known that the potential variation of the system determines the acceleration. The IRD of a carbon atom on the outer ends of the stators directly determines the potential. The second factor is the number of IRD atoms, and is also important because the potential variation is proportional to the number of IRD atoms. The third factor is the length of the stators. Longer stators provide a higher friction force on the rotor. In this simulation there are 7 IRD atoms with the same radial deviation of 0.4 times *L*_c-c_, the bond length between two *sp*^2^ carbon atoms, i.e., 0.142 nm. Hence, we can reduce the acceleration by adjusting the number of IRD atoms and the radial deviation. If we need a longer duration of acceleration of the rotor, we can provide only one IRD atom with lower radial deviation. As the rotor rotates at high speed, the thermal vibration of atoms on it causes non-equilibrium of the rotation. Therefore, the rotor does not rotate in a stable state at a high speed. When one end of the rotor departs the stators, one or more new C-C bonds are formed and the rotational frequency of the rotor becomes zero (see No. 3 insert at 700 ps in Fig. 2a and Movie 1).

Mathematically, the rotational equation can be expressed as:


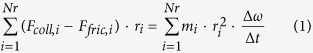


*F*_*coll*,*i*_ is the impact force along the circular direction due to collision of the *i*^th^ atom on the rotor with stators. *F*_*fric*,*i*_ is the friction force along the circular direction on the *i*^th^ atom on the rotor from stators. “*r*_*i*_” is the average radius of the gap between the rotor and stators at the *i*^th^ atom on the rotor. Due to the radial deviation at the ends, the value of *r*_*i*_ is reduced, which is essential to the value of the potential of the system. “*m*_*i*_” is the mass of the *i*^th^ atom on the rotor. Δ*ω* is the increment of angular velocity of the rotor. Δ*t* is the increment of time, which may be 100 times a time step in simulation. “*N*_*r*_” is the total number of atoms on the rotor.

If we add a wing (i.e., GN + end-tube) onto the rotor, the rotational acceleration is far less than that of the pure CNT rotor. For example, the rotational frequency of the rotor increases from 0 to ~48 GHz after ~8000 ps (Fig. 2b). From the configuration of the rotor at 8006 ps, we find that the rotor curves due to the high centrifugal force from the rotating wing. Hence, although the maximal rotational frequency of the rotor is only ~48 GHz, the system fails (see the insert configuration of the system at 8090 ps and Movie 2). Simultaneously, the wing bonded on the rotor also increases the moment of inertia of the rotating part. The acceleration duration of the rotating part becomes higher than that of the pure CNT rotor. If we reduce the number of IRD atoms and the radial deviation, the duration can be extended further.

The above results also imply that the rotation speed of the rotor cannot be measured at such a high speed by traditional *in situ* testing systems. To determine the rotation speed, we suggest providing a slim probe near the trace of the rotating part (i.e., rotor + wing as shown in Fig. 1b–d). In Fig. 2c, the rotational frequency history of the rotor is given when the probe is present and the distance between the probe tip and the axis of the rotor is 10.092 nm. The value is greater than 9.950 nm (the initial radius of the rotating part) and less than 9.950 + 3 × 0.34 nm (beyond the cut-off of L-J interaction between probe and end-tube on wing). Hence, the rotating part will be attracted by the probe tip when the end-tube passes the probe during rotating. From Fig. 2c, we find that the rotating part is in a relatively stable rotating state with the rotational frequency of ~17 GHz (green dashed line in Fig. 2c), or the rotational period of ~58.8 ps. The reason that the rotational frequency of the rotating part does not increase further than ~48 GHz, as shown in Fig. 2b, is mainly the attraction of the probe. This result also indicates that the probe acts as a damper, although that is not relevant to measurement of the rotation of the rotor.

**Figure 3:**
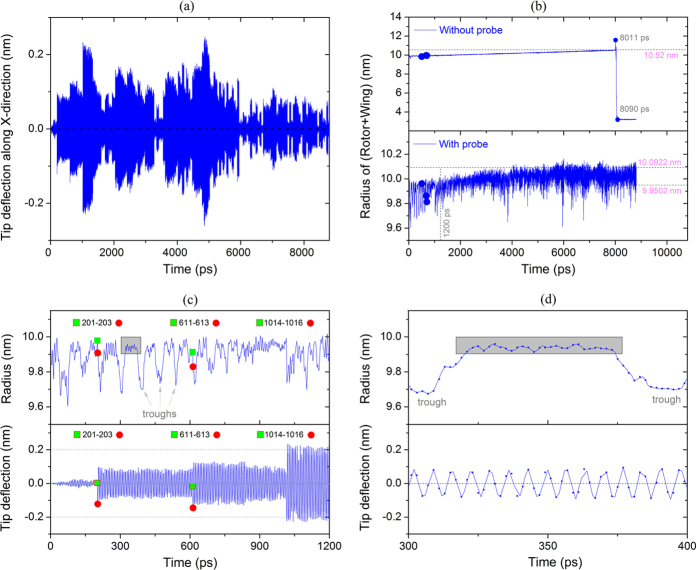
Dynamic responses of rotating part and probe in simulation. (**a**) Deflection history of probe tip along X-direction. (**b**) Radius histories of rotating part, e.g., rotor + wing, with or without probe. (**c**) Radius of rotating part and deflection of probe tip during [0, 1200] ps. (**d**) Radius of rotating part and deflection of probe tip during [300, 400] ps.

Here we want to measure the rotation of the rotor through observing the oscillation of the probe tip due to its collisions with the end-tube in the rotating part. In Fig. 3a, the deflection history of the probe tip (along the X-direction) is shown and the amplitude of oscillation varies sharply. Observing the variation of the length of the rotating part (Fig. 3b) during the period, we find that the radius of the rotating part varies slightly. For instance, the radius of the rotating part is mainly in the interval [9.80, 10.10] nm, unlike the radius of the rotating part in the system without probe (the upper layer of Fig. 3b). This indicates that the potential of the rotating part also changes slightly. Considered together with the slight variation in kinetic energy after ~5000 ps (the rotational frequency shown in Fig. 2c), the conclusion is drawn that the rotation is stable while the probe is present with the distance of 10.092 nm (i.e., *r* + *L*_c-c_, “*r*” is the initial radius of rotational part, e.g., 9.950 nm) between the probe tip and the axis of the rotor in the present model. This conclusion also suggests a new method for controlling the rotational speed of the rotor.

As we observed the radius of the rotating part and the tip deflection during the acceleration period, e.g., [0, 1200] ps, both vary periodically. For example, the value of the radius of the rotating part (the upper layer in Fig. 3c), displays a trough after each ~75 ps. Within each period, the radius varies very slightly (see, for example, the curves in the rectangular gray frame in the period of [320, 370] ps, also see the upper layer in Fig. 3d). When we observe the movies in this period (Movie 3), we find that the slight variation in the rectangular frame is mainly due to the radial oscillation of the rotating part. That is, the GN in the rotating part oscillates like a spring. The troughs in the radius history curve are attributed to the curvature of the GN part being driven to rotate by the rotor. It is known from the above analysis that the rotating part is attracted by the probe tip as it passes by, when the speed of the rotating part decreases sharply. Nevertheless, the speed of the rotor is still high. The difference in rotation between the rotor and the wing leads to the form of curvature of the GN. Therefore, the circular oscillation of the rotating part is triggered with a period of ~75 ps.

**Figure 4:**
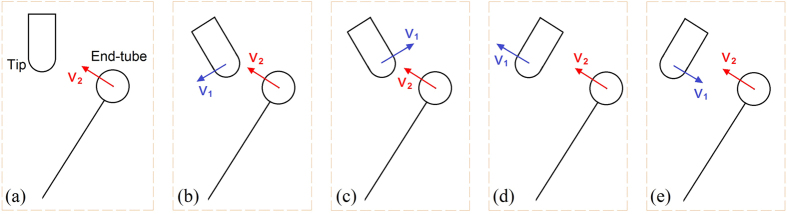
Schematic models of collision between probe tip (with velocity v_1_) and end-tube on wing (with velocity of v_2_). (**a**) Tip stands still or bypasses the equilibrium position. (**b**) Tip returns (to the equilibrium position), followed by end-tube. (**c**) Tip moves away from end-tube. (**d**) Tip moves away followed by end-tube. (**e**) Tip returns against the end-tube. The collision models in (**b**,**d**) can be considered energy-absorption models and the model in (**c**,**e**) can be considered energy-dissipation models.

Actually, the dynamic response of the probe tip is essential for the present measurement. From the lower layer in Fig. 3c, we find that the deflection of the tip has three obvious jumps in the first 1200 ps. The three jumps are near to 200, 610, and 1010 ps, respectively. The amplitudes of oscillation of the probe tip in the three stages are 0.02 nm during [0, 200] ps, 0.09 nm during [610, 1000], and 0.2 nm during [1010, 1200] ps. From the lower layer in Fig. 3d, we know that the probe vibrates with a stable period and the eigen frequency is ~111 GHz. As we observe the movies during the period, we find that each jump in probe tip deflection is due to collision of the tip with the end-tube. The amplitude of tip deflection increases after collisions, implying that the mechanical energy of the probe increases during collision. The reason can be found from the collision models shown in Fig. 4. For example, after the collision between the probe tip and the end-tube at 201 ps, the amplitude of tip deflection increases sharply. The tip deflection at that time is very close to zero. Hence, the collision can be considered consistent with both models in Fig. 4a and b. At 611 ps, the collision also provides mechanical energy to the probe. At that moment, the tip deflection is negative (i.e., the probe tip moves left from its equilibrium position), which means that the collision should be similar to the model shown in Fig. 4d. At 1014 ps, the collision still provides energy to the probe. The tip deflection is positive (i.e., the probe tip moves right from its equilibrium position). Hence the collision model should be similar to that shown in Fig. 4b. However, the deflection history curve shown in Fig. 3a demonstrates that the collision does not always provide energy for the probe. The amplitude of tip deflection drops occasionally if the collision model is similar to those shown in Fig. 4c,e. The duration between two neighboring collisions also varies. That feature occurs mainly because the opportunities for collision models (kinetic energy-absorption model and kinetic energy-dissipation model) are not equal during a particular period of time.

It is also necessary to indicate that the amplitude jump depends not only on the collision model but also on the velocity of the end-tube. If the magnitude of the moment of the end-tube is close to that of the probe, but the velocities are opposite (Fig. 4e), the deflection of the probe tip obviously decreases.

### System with winding wing by hydrogenation

**Figure 5:**
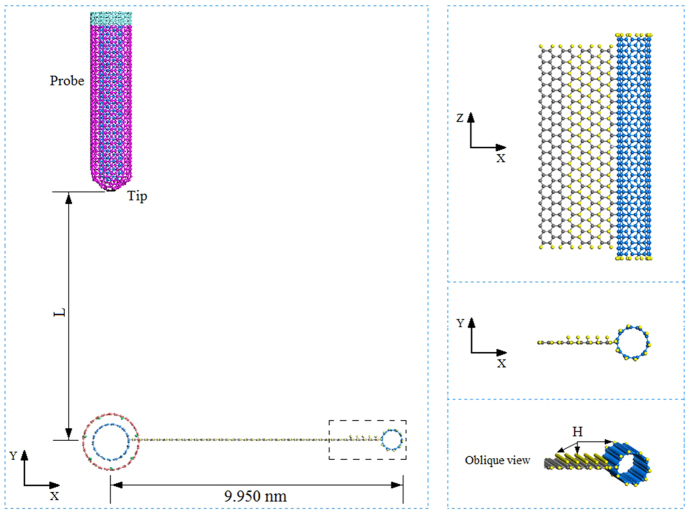
The initial hydrogenated system with new sizes. There are 5 lines (along axis of rotor) of C-H bonds near the end-tube. L is the distance between the probe tip and the axis of the rotor.

From the above simulation, the rotating part has a stable radius during rotation. If the end-tube is initially attracted onto the probe tip, the experiment for testing the rotation of the rotor fails. To overcome this problem, we propose a new rotational part, in which the carbon atoms on the GN and close to the end-tube are bonded with hydrogen atoms. Due to hydrogenation being on one side of the GN, the GN is curved. If there is sufficient hydrogenated area of GN, the end-tube will attract the GN to wind upon it due to L-J interaction. For example, there are 5 lines of carbon-hydrogen bonds near the end-tube in the model shown in Fig. 5^17^,^29^,^32^. Before simulation, we can imagine that the nanoscroll will be unwound if the rotational speed of the rotor is high. The reason is that the centrifugal force applied on the scroll reduces the curvature of GN. As the end-tube collides with the probe tip, the velocity of the rotating part is reduced and the GN winds upon the end-tube again. Hence, the probe tip will have an obvious dynamic response that is be useful for measuring the rotation of the rotor.

### Relationship between the maximal deflection of probe tip and the fixed rotational frequency of wing

In the model shown in Fig. 5, the value of L, the distance between the probe tip and the axis of the rotor, is 8.60 nm, which is less than 9.950 nm of the largest radius of rotating part with fully unwound GN. Before discussing the dynamic response of the rotating part and the probe in the system (in Fig. 5) with the temperature-driven motor, we investigate the characteristics of the oscillation of the probe tip colliding with the rotating part (in Fig. 5) with a constant rotational frequency.

**Figure 6:**
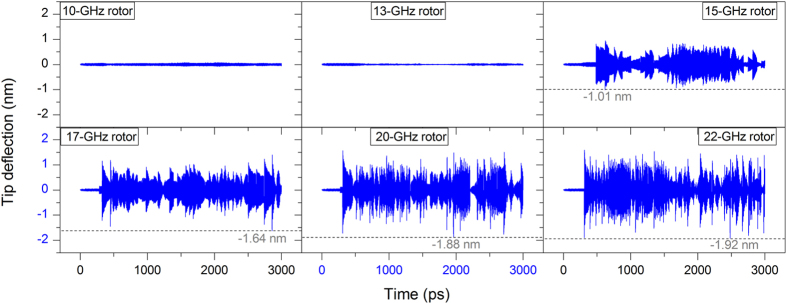
Spectrum of deflection of probe tip colliding with the rotating part with a constant rotational frequency between 10 and 22 GHz when L = 8.60 nm and temperature is 300 K. The peak value of the tip deflection is obtained during 3000 ps of continuous collision with the end-tube.

In Fig. 6, the history of the tip deflection within 3000 ps is given during collision with an end-tube that is driven by the rotor with a constant rotational frequency. When the rotational frequency of the rotor is not more than 13 GHz, the centrifugal force cannot unwind the GN from the end-tube, and no interaction occurs between the probe and the end-tube, due to the small radius of the rotating part. When the rotational frequency of the rotor is not less than 15 GHz, collision occurs and the deflection of the probe tip fluctuates obviously. The peak value of the tip deflection is obtained during 3000 ps. We find that the value increases with the increase of the rotational frequency of the rotor. When the rotor’s rotational frequency is 22 GHz, the maximal amplitude of deflection reaches 1.92 nm, which is ~1/3 the length of the free part of the probe. Hence, the probe tip cannot provide the deflection curve when the rotor has a higher rotational frequency. According to the peak values of the tip deflection, we can estimate the rotational frequency of the rotor that is driven by the stators in the following simulation.

### Local maximal deflection of probe tip v.s. the rotational frequency of wing

**Figure 7:**
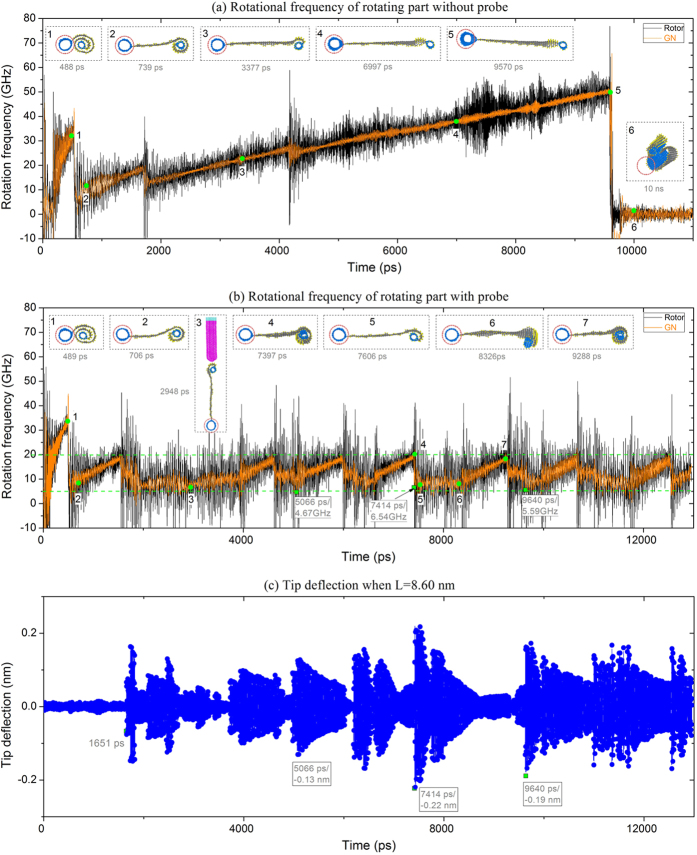
Dynamic response of system with or without probe. (**a**) The rotational frequency histories of the rotor and GN when the system has no probe. (**b**) The rotational frequency histories of the rotor and GN when L = 8.60 nm (<9.9502 nm of radius of rotating part). (**c**) The history of probe tip deflection.

Now, let us return to the original problem, namely measurement of the rotation of a thermal motor with a probe. Figure 7a demonstrates that, without a probe, the rotational frequency of the rotating part (rotor + wing) reaches its maximal value (~48 GHz) after 4 obvious jumps at 300 K. Comparison with the results shown in Fig. 2b shows that here, acceleration of rotational frequency requires a longer time. One reason is the greater number of hydrogen atoms on the present rotating part compared to those in Fig. 2b. Another reason may be the disturbance of the configuration jumps of the rotating part.

In the rotational history of the rotating part, there are six stages. Each stage has different characteristics, as illustrated in the following:

**Stage I**:  During [0, 177] ps, driven by the stators, the rotor just initiates rotating. Simultaneously, the GN ribbon winds upon the end-tube.

**Stage II**: During [178, 529] ps, the rotation of the rotor becomes obvious. When the rotational frequency reaches ~30 GHz the nanoscroll is wound due to centrifugal force. The rotational speed drops to ~5 GHz. As the rotational speed is still too low, a part of the GN remains wound upon the end-tube. (Movie 4).

**Stage III**: During [530, 1698] ps, the rotational speed of the rotor increases continuously before collision. During acceleration, only a small part of the GN is unwound from the scroll. When the rotational frequency of the rotor reaches ~18 GHz, the GN is suddenly unwound from the scroll and the rotational frequency of the rotor jumps for the second time. But this time, the rotational frequency of the rotor is over 10 GHz. As there is still part of the GN wound on the end-tube (see No. 2 insert in Fig. 7a), the radius of the rotating part varies within [8.30, 9.10] nm. If there is a probe in the system, the nanoscroll will be attracted by the probe tip.

**Stage IV**: During [1699, 4162] ps, the rotational frequency of the rotating part increases from ~11 GHz to ~25 GHz before the third jump occurs. As the rotational speed is high, only half of the end-tube is covered by GN. (Movie 5).

**Stage V**:  During [4163, 9585] ps, the rotational speed of the rotating part increases continuously, and the GN flattens. Meanwhile, the transverse deformation of the rotor increases because of the increasing centrifugal force of the rotating part. After 9585 ps, the rotor is pulled out of the stator and the fourth jump occurs.

**Stage VI**: Starting from 9586 ps, the rotor is bonded with one of the stators and the rotating part winds into a scroll. The rotation disappears. (Movie 6).

Figure 7b shows the rotational frequency history of the rotating part when the system contains a probe. In the first 1600 ps, the state of the rotating part is the same as that shown in Fig. 7a, because the radius of the rotating part is still less than 7.6 nm (≈L‒3 × 0.34 nm) and the probe has no influence on the rotating part. After that, the rotational frequency of the rotating part varies nearly periodically between 5 and 20 GHz. For instance, during [7397, 9285] ps, the two configurations of the rotating part vary alternately. At 7397 ps, the rotational frequency of the rotating part is ~18 GHz and the whole end-tube is covered with GN (No. 4 insert in Fig. 7b). When the GN is unwound further, for example, when only half of the end-tube is covered with GN (No. 5 insert in Fig. 7b), the rotational frequency of the rotating part drops to ~9 GHz. As we know, the radius of the rotating part shown in insert No. 4 in Fig. 7b is less than 7.6 nm, which implies that the probe has no influence on the rotating part. But the radius of the rotating part in insert No. 5 in Fig. 7b is greater than 7.6 nm. Hence, the rotation of the rotating part is controlled by the probe tip.

In Fig. 7c, the deflection history of the probe tip is shown when the distance between the probe tip and the axis of the rotor is 8.60 nm. The first sharp jump of deflection occurs at 1651 ps because of the collision of the end-tube with the probe tip. Later, collisions occur frequently. This demonstrates that the amplitude of the tip deflection does not always increase. The damping character of the tip deflection implies that an energy-absorption collision is usually followed by many energy-dissipation collisions. Hence, if we obtain a tip deflection curve with a large number of jumps, we can conclude that the rotor is rotating.

In Fig. 7b,c, the collision results show that the rotational frequency of the rotor at 5066 ps is ~4.67 GHz. At the same time, the amplitude of the tip deflection is ~0.13 nm. At 7414 ps, the rotational frequency of the rotor is ~6.54 GHz and the amplitude of the tip deflection is ~0.22 nm. At 9640 ps, the rotational frequency of the rotor is ~5.59 GHz and the amplitude of the tip deflection is ~0.19 nm. It can be concluded, therefore, that the amplitude of the tip deflection increases after collision, which means that the retained rotational frequency of the rotor is higher, and thus further implies that the rotational frequency of the rotor is higher at the moment the collision occurs. We also find that the amplitude of the tip deflection is far less than that with respect to the “15-GHz rotor” in Fig. 6. That is because the rotational frequency of the rotor varies between 5 and 20 GHz. Due to frequent collisions, the end-tube does not run synchronously with the rotor. Thus the velocity of the end-tube colliding with the tip is lower than that of the end-tube driven by “15-GHz rotor”. Therefore, the present amplitude of the tip deflection is not as obvious as that in Fig. 6.

### Influence of distance between tip and axis of rotor

What happens if we reduce the distance between the probe tip and the axis of the rotor? To reveal the effect of L on the dynamic response of the rotation, in this case, we set L = 8.00 nm. As the value is less than 8.60 nm, the rotating part will be attracted sooner by the probe tip. The rotational frequency of the rotating part will, of course, be different from the frequencies shown in Fig. 7b.

**Figure 8:**
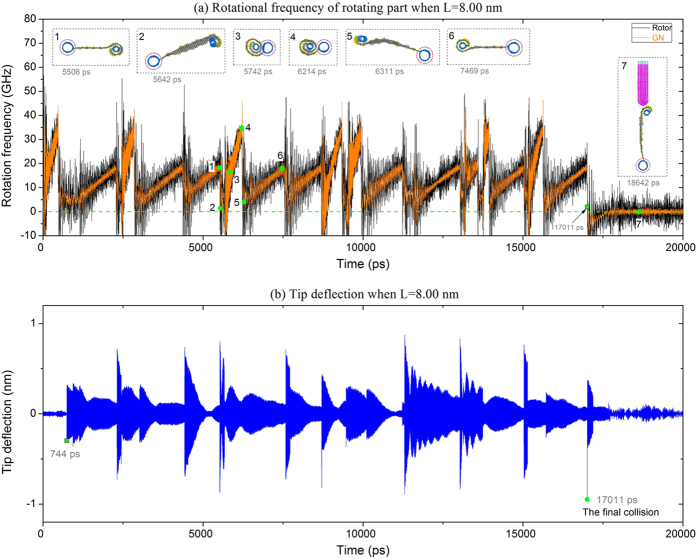
Dynamic response of system. (**a**) Rotational frequency histories of the rotor and GN when L = 8.00. (**b**) History of probe tip deflection.

In Fig. 8a, the rotational frequency history of the rotating part is given when L = 8.00 nm. In the first 529 ps, the rotational state of the rotating part is the same as that of system with L = 8.60 nm (Fig. 7b), because the curved GN is not sufficiently unwound. At 744 ps, the first collision occurs (Fig. 8b). As the distance between the tip and the axis of rotor is less than 8.60 nm, the collision provides higher mechanical energy to the probe. In particular, the first jump of the tip deflection is ~0.30 nm which is higher than ~0.07 nm at 1651 ps in the Fig. 7c (Movie 7). The maximal deflection of the tip even reaches ~0.95 nm at 17011 ps, at which time the final collision occurs. Before rotation ceases, the rotational frequency of the rotating part varies periodically. For instance, during [5508, 7469] ps (Movie 8), the rotational frequency of the rotating part experiences two sudden drops and increases. We also find that the rotational frequencies at 5508 and 7469 ps are approximately equal, and the rotating part also has similar configurations; that is, the end-tube is covered by one layer of GN. The rotational frequency reaches its lowest local value at 5642 and 6311 ps. The configurations of the rotating part are also similar; for instance, only half of the end-tube is covered by GN. The rotational frequency is lower, meaning that a greater amount of the GN is unwound. Hence, it is reasonable that the final kinetic energy-absorption collision occurs at 17011 ps. After that collision, the rotational frequency of the rotating part is negative and the deflection of the probe tip is negative (moves left from its equilibrium position). After 17011 ps, the rotating part is attracted to the probe tip, and both the rotational frequency of the rotating part and the tip deflection tend to be zero (Movie 9).

## Conclusions

To measure the rotation of the thermal-driven motor, we present a system containing a probe and a motor whose rotor has a wing. The wing is made from a GN on an end-tube. When the rotation of the rotor is actuated by the stators with IRD atoms, the end-tube on the wing may collide with the probe tip. Results from the numerical experiments above support following remarkable conclusions: (1) The deflection of the probe tip changes steeply due to collisions with the end tube. The deflection increases if the probe tip and the end-tube have the same direction of motion, or vice versa. (2) When the wing has a fixed rotational frequency, there is a threshold value of the rotational frequency for the occurrence of the collision between the end-tube and the probe tip. This is because the partly hydrogenated wing cannot fully flattened under low centrifugal force and the distance between end-tube and the probe tip is too large. When the rotational frequency of the wing is higher than the threshold value, higher rotational frequency leads to higher value of the maximal deflection of probe tip. When the sizes of elements of the system are known, the threshold is determined. Meanwhile, the maximal rotational frequency of the rotor is also known. Hence, the rotational frequency of the rotor can be estimated using the threshold and the maximal value. (3) If the variation of the deflection of probe tip exists for a short of time and disappears further, the motor is broken due to high value of rotational frequency of the rotor. It means that the distance between the probe tip and the rotary axis of the rotor is higher than the full radius of the rotary part. Moving the probe close to the rotary axis may obtain a stable state of the system.

Currently, the probe made from carbon nanotube has been put into application^31^. A nanobearing from DWCNTs can also be fabricated. Assemble a GN on a tube is a challenge in experiment. Especially, positions of the IRD atoms are difficult to control. When the difficulties above are by-passed, the present method can be used for measuring the rotation of thermal-driven nanomotors.

## Additional Information

**How to cite this article**: Cai, K. *et al*. A method for measuring rotation of a thermal carbon nanomotor using centrifugal effect. *Sci. Rep.* **6**, 27338; doi: 10.1038/srep27338 (2016).

## Supplementary Material

Supplementary Information

Supplementary Information

Supplementary Information

Supplementary Information

Supplementary Information

Supplementary Information

Supplementary Information

Supplementary Information

Supplementary Information

Supplementary Information

## Figures and Tables

**Table 1:** Parameters of the components in the system shown in Fig. 1 (Length unit: nm).

**Component**	**Motor**		**Wing**		**Probe**	
**Name of part**	**Rotor**	**Stator**	**GN**	**End-tube**	**In-tube**	**Out-tube**
Chirality	(9, 9)	(14, 14)	(×, ×)	(5, 5)	(5, 5)	(10, 10)
Length (direction)	~8.116 (*z*)	~0.492 (z)	~4.181 (*z*)	4.673 (*z*)	~5.682(*y*)	~6.026 (*y*)
Diameter/width	~1.220	~1.898	8.378 (*x*)	0.678	0.678	1.356
Number of atoms	1206C*	140C	1400C + 80H	390C + 20H	470C	990C

*“C” represents carbon atom; “H” represents hydrogen atom.
