# Supplementary material for: A method for measuring rotation of a thermal carbon nanomotor using centrifugal effect
Source: Sci Rep. 2016 Jun 2;6:27338. doi: 10.1038/srep27338 (PMC4890290; doi:10.1038/srep27338)
Supplement: Supplementary Information [file srep27338-s1.pdf]

A method for measuring rotation of a thermal carbon nanomotor using centrifugal effect

Kun Cai [a, b], Jingzhou Yu [a], Jiao Shi [a], Qing H. Qin [b, *]

[a] *College of Water Resources and Architectural Engineering, Northwest A&F University, Yangling 712100, China*
[b] *Research School of Engineering, the Australian National University, Canberra, ACT, 2601, Australia*
* Corresponding author's email address: qinghua.qin@anu.edu.au (Qinghua Qin)

Supplementary information accompanies this paper:

Movie 1. Video for illustrating Fig.2a during [650-750ps]
Movie 2. Video for illustrating Fig.2b during [7901-8900ps]
Movie 3. Video for illustrating Fig.3c during [200-600ps]
Movie 4. Video for illustrating Fig.7a during [450-700ps]
Movie 5. Video for illustrating Fig.7a during [1650-1850ps]
Movie 6. Video for illustrating Fig.7a during [9500-9800ps]
Movie 7. Video for illustrating Fig.8a during [0-3000ps]
Movie 8. Video for illustrating Fig.8a during [4000-7000ps]
Movie 9. Video for illustrating Fig.8a during [16800-18000ps]
